# From Open Access to Circular Health: Ilaria Capua’s Journey through Science and Politics

**DOI:** 10.3390/v14061296

**Published:** 2022-06-14

**Authors:** Elisa Crisci

**Affiliations:** Department of Population Health and Pathobiology, College of Veterinary Medicine, North Carolina State University, Raleigh, NC 27607, USA; ecrisci@ncsu.edu; Tel.: +1-9195136255

**Keywords:** Ilaria Capua, open access, circular health, one health, influenza virus

## Abstract

This paper highlights the career of an exceptional woman virologist, Dr. Ilaria Capua. It recollects her major achievements, awards and noteworthy events that have shaped her scientific and political career. It retraces Dr. Capua’s major contributions to the study of viral zoonoses, in particular influenza virus, and her strong commitment to an open, more ethical science at the service of society in its broadest sense. It describes how she became the long-term champion of “Open Access” and “Data Sharing” for virus genetic sequences and introduces her new concept of “Circular Health”, where health becomes a circular system that represents a central and vital connection hub between humans and nature. This paper features Dr. Capua’s value as a role model for young women scientists and their empowerment.

## 1. Introduction

**Figure 1:**
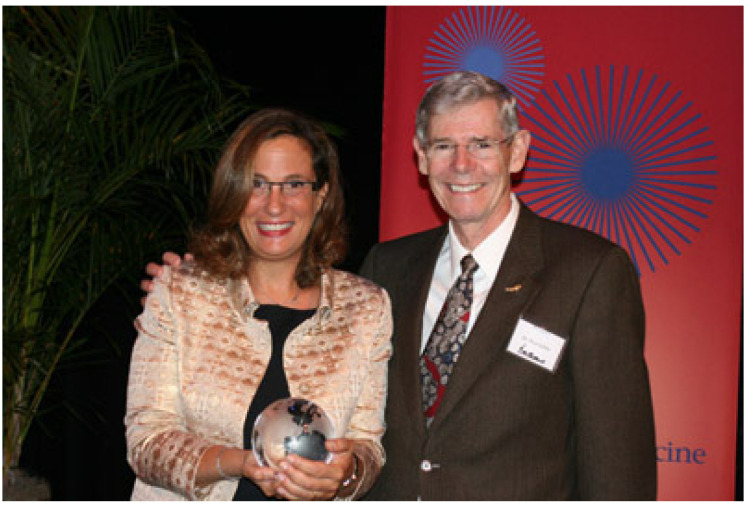
Dr. Ilara Capua and Dr. Paul Gibbs during the ceremony for the Penn Vet World Leadership in Animal Health award conferred by the University of Pennsylvania (USA) in 2011.

This paper retraces the journey of an outstanding veterinary virologist—a woman with a forward-looking mind, promoting paradigm-shifting ideas, a woman whose words carry weight [1]—Dr. Ilaria Capua (Figure 1).

Following over two decades working in virology diagnostic and research laboratories, she became a “politically modified organism” [2] when she joined the Italian parliament to promote a more meritocratic and international approach to Italian research, bringing to the political discussion table the One Health approach with particular focus on emerging infectious diseases and antimicrobial resistance. Incredibly, during her time in parliamentary office, she was accused of being an international “virus trafficker” who was deliberately creating epidemics of avian influenza for personal economic advantages [3]. She was shamed by media, virtually ignored by the scientific community, insulted and offended by some of the opposition parliamentarians, and threatened with life imprisonment [2,4]. She had to fight to regain her reputation against a mass media system that often seeks to criminalize and discredit individuals before the truth is established by a court of law [2,4]. “She was able to see through the fog” [4], she proved her innocence against mendacious accusations, she started over again, showing that what really matters is to be resilient, and “challenges are opportunities to understand it is time for a change” [2,4]. Since 2016, she has served as pre-eminent professor and Director of the One Health Center of Excellence at the University of Florida (https://onehealth.ifas.ufl.edu/about/one-health-center-of-excellence-team-directory/ilaria-capua-profile/ accessed on 6 June 2022).

I hope her story will empower future women virologists to fight for the recognition of their professional accomplishments and for their careers, to be brave and to promote their ideas, regardless of the challenges they encounter.

## 2. Who Is Dr. Ilaria Capua?

Dr. Capua studied veterinary medicine at the University of Perugia and earned two post-graduate degrees: the first one at the University of Pisa (1991, equivalent to a Master’s degree), and a Ph.D. from the University of Padua (2007), in virology and public health, focusing her dissertation on avian influenza.

**Figure 2:**
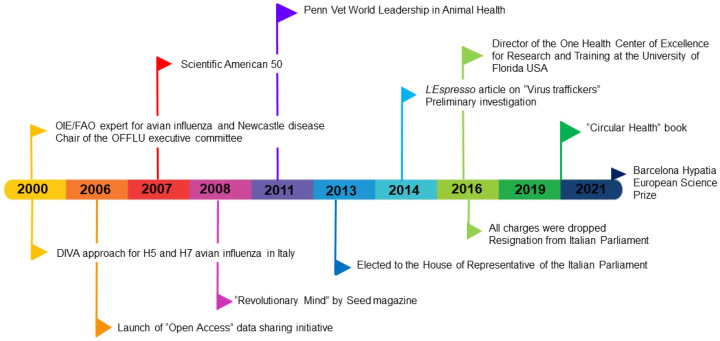
Timeline of Dr. Ilara Capua’s career from 2000 until 2021.

She worked in different national diagnostic and research institutes, part of the network of Istituti Zooprofilattici Sperimentali (IZS), until she reached the IZS of Padua, where she became director of the Division of Comparative Biomedical Sciences and director of the FAO/OIE and National Reference Laboratory for Avian Influenza and Newcastle Disease. In Padua, she reached the peak of her international recognition with her work on avian influenza viruses (selected publications [5,6,7,8,9,10,11,12,13,14,15,16,17,18,19]) and actively promoted the One Health concept [20], even outside the biomedical community. At IZS Venezie, she became the long-term champion of open access to genetic data on pre-pandemic and pandemic viruses [21]. She was described in a profile in Science as “Italy’s Influenza DIVA” [1] for her charisma, strong personality and the development of a DIVA vaccination system for avian influenza in poultry [22]. That DIVA (Differentiating Infected from Vaccinated Animals) approach successfully controlled epidemics of notifiable H5 and H7 avian influenza subtypes between 2000 and 2006 in Italy [22] (Figure 2).

Not surprisingly, her major scientific impact was during her years in Padua, a city called by Herbert Butterfield the birthplace of scientific revolution [23]. There, she received the nickname “the lady of viruses” [2]. Thanks to her international recognition and expertise, she was the nominated OIE/FAO expert for avian influenza and Newcastle Disease and was among the initiators and chaired the executive committee of the OFFLU network (OIE-FAO network of expertise on animal influenza) [24] (Figure 2), which, in support of countries affected by the avian influenza virus, promotes the exchange and analysis of influenza virus strains and represents one of the links between the veterinary community and certain organizations belonging to the United Nations network.

Dr. Capua worked tirelessly to encourage Italian excellence in science and to empower young women scientists, leading by example and accomplishing outstanding work despite hardships caused by chronic underfunding of Italian research institutes, antimeritocratic environments and scarce attention to gender balance at that time [1,25]. Indeed, she was a female veterinarian operating in a heavily male-dominated field. A veterinarian, a woman, and a virologist: a combination of three “dangerous” words that positioned her in the eye of a large storm that was ahead [2]. She was thinking ahead of others and was determined to openly express her ideas.

## 3. Dr. Capua, Pioneer in the Concept of Data Sharing for Avian Influenza Viruses (2006–2012)

In the COVID-19 era, “open science” and “open access“ principles have been the foundation for scientific advancement and for the implementation of public health measures as a response to the pandemic. Since 2020, sharing information and communicating new scientific discoveries have occurred at an unprecedented pace; accessible unrestricted knowledge is now taken for granted because it maximizes the discovery of new weapons to fight against the emerging pandemic. Social media platforms are a tool to communicate science and public health measures, although they have also led to stirring up anti-science movements. Preprints have become the standard to showcase new findings, and scientific collaboration has led to incredible discoveries in an accelerated manner that have flooded the press worldwide. This pathway has changed science forever [26].

Until recent times, however, data sharing and open access databases were more often the exception than the rule, with scientists fighting to protect their data before publishing or patenting [27,28]. At the turn of the millennium, leading laboratories working with emerging pathogens, such as avian influenza viruses, deposited their sequences in password-protected databases accessible only to a very limited number of users [28]. In this setting, Dr. Ilaria Capua became the pioneer in a new data sharing approach for genetic sequences of zoonotic viruses. She ignited an international debate and was outspoken in advocating the sharing of information on avian influenza virus [21], which causes deadly epidemics in avian species, and given its zoonotic potential, is a continuous threat to human health [29,30].

In 2006, at the peak of the H5N1 epidemic in Europe, Asia and Africa, Dr. Capua was a leading influenza scientist at the Istituto Zooprofilattico Sperimentale delle Venezie (Italy) and was internationally recognized for her work in the lab and in the field. Together with her team, she decided to deposit an unpublished genetic sequence of a Nigerian H5N1 avian influenza virus in a public database (GenBank accession number: DQ406728.1) [14,31] instead of entering the information into a password-protected database (Figure 2). She was very vocal about this deliberate choice; in her view, it was essential to address a major health threat and improve pandemic preparedness and response. To make this effort more powerful, she sent a message to the scientific community asking others to follow suit (her posting won ProMED’s annual award in August 2006) [1,28] (Figure 2), leading international veterinary virologists to share their data [31]. These were the first steps to initiate an international debate at the World Organisation for Animal Health (OIE) headquarters on the need for sharing avian influenza virus sequences in an open access environment, to boost research and preparedness in a pre-pandemic phase [32]. This new movement challenged the existing paradigm on data sharing across disciplines and organizations that had resulted from fear of unpublished data being scooped [1,28].

Dr. Capua’s vision was embraced by a wider group of scientists, including Nobel laureates and stakeholders, for the expansion and establishment of new data sharing organizations as the influenza genome sequencing project [33], the Global Initiative on Sharing Avian Influenza Data (GISAID) [34,35] and the Influenza Research Database (IRD. CEIRS program, https://www.fludb.org/brc/home.spg?decorator=influenza accessed on 6 June 2022) [36], all with the aim of setting up an open access system for sharing influenza virus sequences. Nowadays, GISAID is used for influenza viruses worldwide and more recently, it has become a popular genome site for coronavirus sequences, hosting more than 10 million sequences in 2022 (https://www.gisaid.org/, accessed on 4 May 2022) [35].

## 4. Italian Parliament Experience and Criminal Investigation (2013–2016)

In 2013, Dr. Capua was at the pinnacle of her scientific carrier, recognized nationally and internationally for her expertise in the field of virology and for her strategic leadership in science. In addition, she started authoring books for the general public, which expanded her reach beyond the scientific community. Thanks to her increased visibility in the Italian media, she had the opportunity to consider a new direction for her career: politics. The Italian Prime Minister in office at the time, Mario Monti, asked her to run for a seat in the parliament as “a member that can understand the complexity of science policy and can defend and promote science to make Italy more competitive” [3]. It was a 24 h decision; her will to promote science and to empower more women scientists helped her to make this leap of faith [2].

She was elected to the House of Representatives of the Italian Parliament and then elected as Vice Chair of the 7th Science, Culture and Education Commission of the Chamber of Deputies of the Italian Parliament [3] (Figure 2). She was responsible for three years in a row for allocating national funding to Italian research institutions. She also championed and achieved property tax-exempt status on buildings housing research institutions.

But, it was not a warm “welcome to politics”. From the early stage of her mandate, she had to endure numerous challenges. She felt lost in the political environment, with a mixture of astonishment, indignation and anger [2]. In addition, a few months after her election in 2013, her father passed away [2]. She was already disoriented, trapped in an alienating place, far from the work she loved, and under baseless and exploitative attacks [2], but much worse was yet to come. One year after her election, an Italian weekly magazine, *L’ Espresso,* published a cover article entitled, “Virus traffickers: scientists have agreements with ‘Big Pharma’ to sell their vaccines and creating epidemics” [2,37,38] (Figure 2). The article was based on the summary of a classified investigation that was leaked to the press [2,3,4]. The initiative to share genetic data was misunderstood by investigators, and she was accused of being the criminal mastermind behind an organization that was selling influenza field viruses to pharmaceutical companies to enable them to produce vaccines for circulating avian strains [2,3,4]. Furthermore, they alleged that there was evidence of deliberate spread of pathogenic viruses into the environment, with the criminal intention of establishing avian influenza epidemics in poultry and people and in Italy between 1999 and 2008 [2,3,4].

Dr. Capua learned about the accusations from a weekly magazine that published the article. Until then, she had no idea that she was under such an investigation, nor would she have had any reason to even consider such a possibility [2]. The article was entirely grounded on the misunderstanding of scientific facts and unfounded theories of a conspiracy between scientists and Big Pharma. After the public scandal, Dr. Capua was stuck in a judicial system that was slow and ruthless [2,4]. She was shamed in the media and violently attacked whilst in office at the Italian parliament. She was interrogated by populist parliamentarians and asked publicly and repeatedly to resign [3]. The parliament was not supporting her, there was no solidarity except for a few colleagues and friends. She was abandoned by the government, the mass media and a portion of the scientific community, which preferred to look away [4]. Her career in science appeared to be over, and her international reputation became a national shame [2].

It took about 2 years and 400 written pages to defend her professional work and reputation, and to fight a highly bureaucratic and scientifically incompetent judiciary system, to prove that she and her team had nothing to do with virus trafficking [2,3]. Over two years after the cover-page article, the judge for the preliminary investigation reviewed the case and dropped all charges against her and her team because “there was no case to answer as the facts reported in the investigation never happened.” [2,3].

In 2016, after being completely cleared [39], Dr. Capua resigned from parliament [2,3] (Figure 2). Her resignation speech focused on the science/justice interface and has been viewed by several millions [40].

Sometimes, wounds caused by a legal proceeding are incurable, and certain types of pain can never be overcome (Prof. Severino [4]). Luckily, Dr. Capua was extremely resilient and able to pick herself up, but she needed to put “an ocean between herself and her own country” [2,4]. Indeed, she moved to the other side of the Atlantic, and became the Director of the One Health Center of Excellence for Research and Training at the University of Florida, USA (Figure 2).

## 5. The New Concept of “Circular Health” and Current Role

In Florida, Dr. Capua has been very active in promoting the “One Health r-Evolution”, which capitalizes on the One Health approach that was developed until then [41]. She reviewed and elaborated on how our knowledge about health has led us to embrace the One Health concept. However, in this work, she foresees and advocates for an inevitable expansion of the One Health concept into a more inclusive approach that exploits contemporary opportunities and is flexible enough to incorporate current and future challenges [41]. This seminal essay laid the foundations for the development of the “Circular Health” concept (Figure 2) [42]. The book “Circular Health: Empowering the One Health Revolution” is a journey through science, infectious diseases and the relationship between science and politics [23,43]. In this book, she unveils how novel interconnections between human, animal and environmental health were uncovered over the centuries and were often refuted by the scientific community of the time. Her vision is that as scientists working at the interface between disciplines, we should always maintain an open mind to new discoveries and should not fear to expand knowledge.

In the book, Dr. Capua continued to highlight the need for sharing knowledge and data, remarking that this is even more important now that scientists are immersed in the big data environment [23]. After all, her initiative on sharing genetic sequences in the face of the avian H5N1 crisis paved the way for an open data sharing that we take for granted now during the COVID-19 pandemic.

Thanks to her drive for change, we can now count on a different mindset that has yielded millions of shared viral sequences studied in real-time. This availability of enormous amounts of virological data can be exploited in a real pandemic, and can be combined with the availability of other real-time data (e.g., Our World in Data (https://ourworldindata.org accessed on 6 June 2022), The New York Times, WHO situation reports etc.). Having accessible large datasets has shown to be a transformational resource during these years of crisis.

The COVID-19 pandemic has clearly shown the world that that human health can no longer be the sole primary objective of decision makers, but that we must address health as a system, recognizing that the health of humans and other animals, plants and the environment are interconnected, interdependent and linked to one another by microbes. However, for example, also pollutants, pharmacologically active compounds and climate change are connectors between the health of humans, animals, plants and environment [23]. Social tools have become “health drivers” and misinformation through media channels has demonstrated that people can be easily misled and motivated to do the exact opposite of what is needed during the early stage of a pandemic. We know that politics have influenced health outcomes since the time of Hippocrates and, for many centuries and for a variety of different reasons, scientists have been insulted, reviled, mocked and persecuted when they stood up against the accepted theories of the time, often paying personal prices for defending their ideas [23].

Dr. Capua continues to be a role model for young women scientists, and even more for veterinary virologists. She has invested her time writing books for the general public and for children [2,23,43,44,45,46,47,48,49,50,51]. She often participates in radio and TV shows with the aim of making science accessible to lay people and of promoting the empowerment of women in science. One of her favorite quotes is that “knowledge is a sustainable resource: using and sharing knowledge never exhausts it but is the road to its multiplication” [23]. For her leadership role in sharing information internationally, Dr. Capua was named among the Scientific American 50 (world’s top 50 researchers for leadership in science policy) in 2007 [52] (Figure 2), and heralded as a “Revolutionary Mind” by Seed magazine in 2008 [53]. She received the most prestigious award in veterinary medicine, the Penn Vet World Leadership in Animal Health Award, from the University of Pennsylvania (USA) in 2011 (Figure 1 and Figure 2) [53].

In 2021, Dr. Capua received the Barcelona Hypatia European Science Prize from the Academia Europaea (Barcelona Knowledge Hub) [54] (Figure 2) for her major contribution to the study of viral zoonoses, for her strong commitment to an open, more ethical science at the service of society in its broadest sense, and for her value as a role model for young women scientists. She dedicated the award to “all the women who have silently made this pandemic much less devastating than it could have been: taking care of other people, working behind the scenes in laboratories. To all those women who have contributed to science and never been recognized” [54].
